# Extract of *Ephedra sinica Stapf* Induces Browning of Mouse and Human White Adipocytes

**DOI:** 10.3390/foods11071028

**Published:** 2022-04-01

**Authors:** Se-Jun Park, Dong-Hyun Shon, Yang-Hwan Ryu, Yong Ko

**Affiliations:** 1 Division of Biotechnology, College of Life Sciences and Biotechnology, Korea University, 145, Anam-ro, Seongbuk-gu, Seoul 02841, Korea; sejun_park@korea.ac.kr (S.-J.P.); ddiyong50@gmail.com (D.-H.S.); brain1905@hanmail.net (Y.-H.R.); 2 NMS Labs, 311, Anyang-ro, Manan-gu, Anyang-si 14001, Korea; 3 R&D Institute, Biosolution Co., Ltd., 232, Gongneung-ro, Nowon-gu, Seoul 01811, Korea

**Keywords:** adipocyte browning, *Ephedra sinica Stapf*, mitochondria, obesity, thermogenesis, UCP1

## Abstract

Browning of adipocytes using herbal extracts is an attractive and realistic strategy for obesity treatment. *Ephedra sinica Stapf* (*E. sinica*) is an Asian traditional medicine known to activate brown adipocytes. To evaluate the effect of *E. sinica* (EEs) on the browning of white adipocytes, expression levels of browning markers, including uncoupling protein 1 (UCP1), were determined using qPCR, Western blot, and immunocytochemistry after mature mouse inguinal preadipocyte (mIPA) and human adipose-derived stem cells (hADSCs) were treated with EEs. In addition, mitochondrial activity was determined by analyzing MitoTracker staining, mtDNA copy number, and oxygen consumption rate (OCR). Treatment with EEs suppressed lipid accumulation and expression levels of adipogenic markers, including *Pparg*, during mIPA differentiation. In mature mIPA and hADSCs browning markers, including *Ucp1*, were up-regulated by EEs. In addition, EEs increased expression of mitochondrial genes, mtDNA copy number, and OCR. EEs showed a dual function: inhibiting adipogenesis in immature preadipocytes, and promoting thermogenesis via browning in mature white adipocytes. Therefore, *E. sinica* is a potential herb for regulating energy metabolism by inducing the browning process.

## 1. Introduction

Obesity is a worldwide disease. Its incidence has increased steadily in recent years. The consequences of obesity are threatening due to the side effects of several metabolic diseases including type 2 diabetes, heart disease, hypertension and hyperlipidemia [1]. The pathogenesis of obesity involves an imbalance between energy intake and expenditure that causes accumulation of excess energy in adipose tissues in the form of triglycerides. Therefore, available strategies for treating obesity include reducing energy intake and increasing energy expenditure [1,2].

There are three types of adipocytes in the body that play a crucial role in energy homeostasis and endocrine action: white, beige, and brown adipocytes [3]. Whereas white adipocytes mainly store energy, beige and brown adipocytes expend energy through non-shivering thermogenesis, which is considered a potential treatment strategy for obesity [3,4]. The thermogenesis of beige and brown adipocytes can dissipate stored energy as heat through uncoupling protein 1 (UCP1), which uncouples ATP production in mitochondrial respiration and regulates the concentration of protons, producing heat [5]. Although these two types of adipocytes (beige and brown) have similar biochemical and physiological characteristics, they are differentiated through different developmental pathways [6]. Beige adipocytes can be converted from white adipocytes by various stimuli. This process is known as browning. The browning of white adipocytes is induced substantially by specific environmental, genetic and pharmacological stimuli [7]. Browning of white adipocytes promotes mitochondrial biogenesis and activity, along with UCP1 expression, enabling energy expenditure, such as classical brown adipocytes [8]. One of the most representative physiological triggers in this process is norepinephrine, an agonist of the β3-adrenergic receptor (ADRB3) secreted after stimulating the sympathetic nervous system by chronic cold exposure [9]. Various other factors, including neuronal factors, the microbiome, immune cells, and several hormones, can also activate adipocyte browning in mice [10,11,12]. In addition, it has been recently reported that several herbal drugs extracted from plants can promote the browning of adipocytes and prevent obesity [13,14].

*Ephedra sinica Stapf* (*E. sinica*), also known as Ma-Huang, is a herbal extract that has been utilized in traditional Asian medicine to treat asthma, coughs and fever [15]. Recent studies have reported that *E. sinica* has anti-arthritic, antioxidant, anti-inflammatory, anti-obesity and neuroprotective effects [16,17]. Ephedrine is the principal active constituent of *E. sinica* and is a sympathomimetic agonist at adrenergic receptors [15]. Extract of *E. sinica* (EEs) is effective in increasing the resting metabolic rate and in losing weight in obese humans [18]. Moreover, several studies have reported that ephedrine can inhibit lipid accumulation in mouse 3T3-L1 adipocytes and activate thermogenesis in brown adipocytes [19,20,21]. All these findings lead us to hypothesize that *E. sinica* has a positive effect on obesity through adipocyte browning. Therefore, the objective of this study was to investigate the underlying mechanisms involved in the biological effects of EEs in the browning of mature white adipocytes using mouse and human primary white adipocytes.

## 2. Materials and Methods

### 2.1. Preparation of Ephedra sinica Stapf Extract

*E. sinica* was purchased from Dongwoodang Pharmacy Co., Ltd. (Yeongcheon, Gyeongsangbuk, Korea). Grounded *E. sinica* was extracted with distilled water under reflux at 80 °C for 3 h. The extract was filtered with gauze, evaporated, and freeze-dried under a vacuum. EEs was stored at −70 °C until further use. It was dissolved in dimethyl sulfoxide (DMSO) at a concentration of 100 mg/mL for experiments.

### 2.2. Cell Culture

Mouse inguinal preadipocytes (mIPA) were obtained from inguinal white adipose tissues of 10-day-old mice. Isolated mIPA cells were cultured in a 1:1 mixture of Dulbecco’s modified Eagle’s medium and Ham’s F-12 (DMEM/F12) containing 1% penicillin-streptomycin, 20% fetal bovine serum (FBS) and GlutaMAX (all from ThermoFisher, Waltham, MA, USA) at 37 °C in a humidified atmosphere with 5% CO_2_ [22]. For mIPA differentiation, cells were cultured to confluence (day 0) DMEM/F12 containing 1% penicillin-streptomycin, 10% FBS, and GlutaMAX (growth medium) and then induced to differentiate with an induction medium supplemented with 0.5 mM isobutylmethylxanthine (IBMX), 1 µM dexamethasone, 1 µM rosiglitazone, and 5 µg/mL of insulin for 2 days (Sigma-Aldrich, St. Louis, MO, USA). They were further cultured for 6 days until maturation completed in the maintenance medium composed of a growth medium containing 5 µg/mL of insulin.

Human adipose-derived stem cells (hADSCs) were obtained from Biosolution Co., Ltd. (Seoul, Korea). These hADSCs were grown in growth medium at 37 °C in a humidified atmosphere with 5% CO_2_. They were differentiated into mature adipocytes after modifying the protocol of Lee and Fried [23]. The adipogenic induction medium was supplemented with 0.5 mM IBMX, 100 mM dexamethasone, 1 µM rosiglitazone, 100 nM insulin and 1 µM 3 3′5-triiodo-L-thyronine (T3) (Sigma-Aldrich, St. Louis, MO, USA). The induction medium was replaced every 2–3 days for 7 days. Maintenance medium containing 100 nM insulin and 1 µM T3 was then provided every 2–3 days for 8 days until adipogenic differentiation was completed (day 15).

### 2.3. Cell Viability Assay

mIPA and hADSCs were seeded into 96-well plates at a density of 5 × 10^3^ cells/well. After 24 h, cells were incubated with culture medium in the presence of EEs for 48 h and DMSO was used as control. Cell viability was assayed using an EZ-Cytox assay kit (Dogen, Korea). The absorbance was measured at 450 nm using a PowerwaveTM XS microplate spectrophotometer.

### 2.4. RNA Isolation and Reverse Transcription Quantitative PCR

Total RNA was isolated from cultured cells using a TRIzol reagent (ThermoFisher, Waltham, MA, USA). After RNA quantification, cDNA was synthesized using 1 µg of total RNA and a cDNA synthesis kit (CellSafe, Korea), according to the manufacturer’s instructions. PCR primers are described in Table S1. Quantitative PCR (qPCR) was performed with a StepOnePlus Real-Time PCR system (ThermoFisher, Waltham, MA, USA) using a Premier qPCR kit (NanoHelix, Korea).

### 2.5. Western Blotting Analysis

Cells were lysed in RIPA lysis buffer (Bio Basic Inc., Markham, ON, Canada). A total of 20 µg protein was separated by electrophoresis on 12% SDS-polyacrylamide gels and transferred to PVDF membranes (GE Healthcare, Chicago, IL, USA). These membranes were then blocked with 3% skimmed milk and probed with primary antibodies (anti-beta actin, anti-PGC1 alpha and anti-UCP1 from Santa Cruz Biotechnology, Dallas, TX, USA). Membranes were washed with TBS-T buffer and incubated with horseradish peroxidase-conjugated secondary anti-rabbit antibodies (Santa Cruz Biotechnology, USA). Finally, signals were detected with an enhanced chemiluminescence system (ATTO, Amherst, New York, NY, USA) using an ImageQuant LAS 4000 mini (GE Healthcare, Chicago, IL, USA).

### 2.6. Oil Red O Staining

Cells were washed three times with PBS and fixed with 4% paraformaldehyde for 30 min at room temperature. These cells were stained with a 0.5% filtered Oil Red O (ORO) solution (Sigma-Aldrich, St. Louis, MO, USA) in 60% isopropanol for 1 h at room temperature and then washed three times with PBS. To extract the incorporated ORO dye, 100% isopropanol was added to the stained cell culture plate. The plate was shaken for 5 min at room temperature. Colorimetric analysis was then performed at 490 nm using a PowerwaveTM XS microplate spectrophotometer. Cell images were detected with a microscope (CKX41, Olympus, Tokyo, Japan) with digital camera (UCMOS05100KPA, ToupTek, Hangzhou, Zhejiang, China).

### 2.7. MitoTracker Staining

To measure changes in mitochondria number upon EEs treatment, staining of fully differentiated adipocytes in 37 °C pre-warmed staining solution containing 200 nM MitoTracker Red probe (ThermoFisher, Waltham, MA, USA) was added into mature adipocytes for 40 min at 37 °C in a humidified atmosphere with 5% CO_2_. Stained images were detected with a fluorescence microscope (IX71) with digital camera (DP71) (both from Olympus, Tokyo, Japan).

### 2.8. Immunocytochemistry

Fully differentiated adipocytes were fixed with 4% paraformaldehyde for 15 min, washed three times with PBS, permeabilized and blocked with PBS containing 0.3% Triton X-100 and 3% BSA for 45 min. These cells were incubated with anti-UCP1 antibody (ABclonal Inc., Woburn, MA, USA) (diluted 1:300) at 4 °C overnight. After PBS washing, they were incubated with Alexa Fluor 488 secondary antibody (diluted 1:500) for 90 min at room temperature in the dark. Cell images were detected with a fluorescence microscope (IX71) with digital camera (DP71) after 4′,6-diamidino-2-phenylindole (DAPI) (Vector Laboratories Inc., Burlingame, CA, USA) staining.

### 2.9. Mitochondrial DNA Copy Number Determination

Total DNA of differentiated adipocytes was isolated using a DNA kit (Bioneer, Daejeon, Korea) according to the manufacturer’s instructions. qPCR was performed and analyzed for mitochondrial DNA copy number based on the relative expression ratio of mitochondrial-encoded gene (mt-RNR2) and nuclear-encoded gene (B2M).

### 2.10. Oxygen Consumption Rate

mIPA cells were plated in Seahorse XF 24-well microplates and fully differentiated for 6 days. After incubation with 10 µg/mL of EEs for 6 h, mitochondrial stress test was performed by treating cells with 2 µM of oligomycin, 1 µM of FCCP, and 1 µM of rotenone/antimycin A, sequentially followed by measurement of energy metabolism of live cells using an XF24 Analyzer (Agilent, Santa Clara, CA, USA), according to the manufacturer’s instructions.

### 2.11. Statistical Analysis

Results are presented as means ± standard error of the mean (SEM). Differences between groups were analyzed by one-way analysis of variance (ANOVA), followed by Tukey’s post hoc test, using a GraphPad Prism 6 (San Diego, CA, USA). *p* < 0.05 was considered significant.

## 3. Results

### 3.1. EEs Inhibits the Adipogenic Differentiation of mIPA

**Figure 1:**
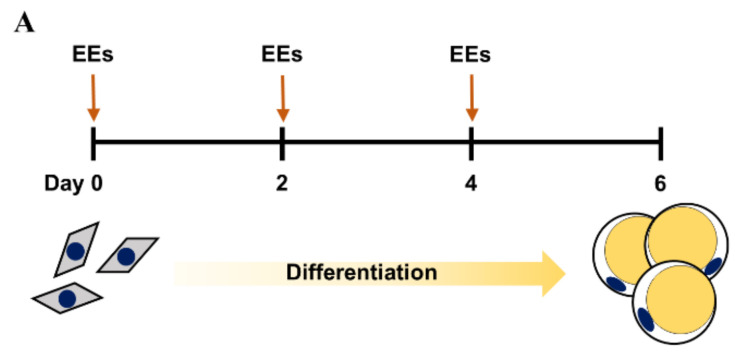

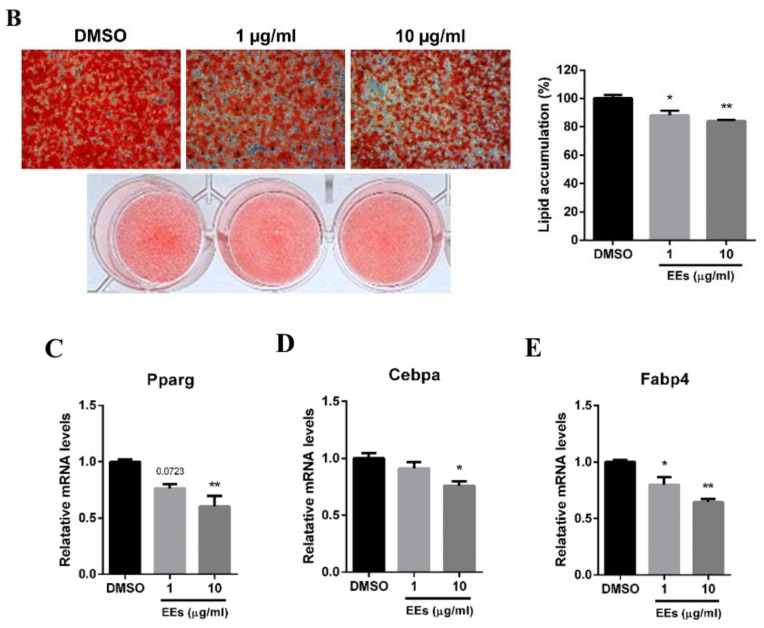
Effects of EEs on differentiation of mIPA. (**A**) mIPA differentiation was induced with differentiation media containing EEs every 2 days until day 6. (**B**) Differentiated cells were treated with EEs and stained with ORO. ORO dye was extracted with 100% isopropanol and absorption was measured at 490 nm. (**C**–**E**) Expression levels of *Pparg*, *Cebpa*, and *Fabp4* were assayed by real-time qPCR. Results are shown as mean ± SEM (*n* = 3). *, *p* < 0.05; **, *p* < 0.01 compared to the untreated control group.

Non-cytotoxic concentrations of EEs in mIPA were observed at 1 and 10 μg/mL through cell viability assays (Figure S1A). To study the effect of EEs on the differentiation of mouse white adipocytes, cells were treated with EEs (1 and 10 μg/mL) every two days for six days during mIPA differentiation (Figure 1A). ORO staining of differentiated mIPA showed significantly decreased lipid accumulation in the 10 μg/mL EEs-treated group compared to the untreated control group (Figure 1B, *p* < 0.01). Additionally, expression levels of adipogenic genes, including *Pparg*, *Fabp4* (*p* < 0.01), and *Cebpa* (*p* < 0.05), were inhibited by EEs in a dose-dependent manner (Figure 1C–E).

### 3.2. EEs Induces the Browning Process in Differentiated mIPA

**Figure 2:**
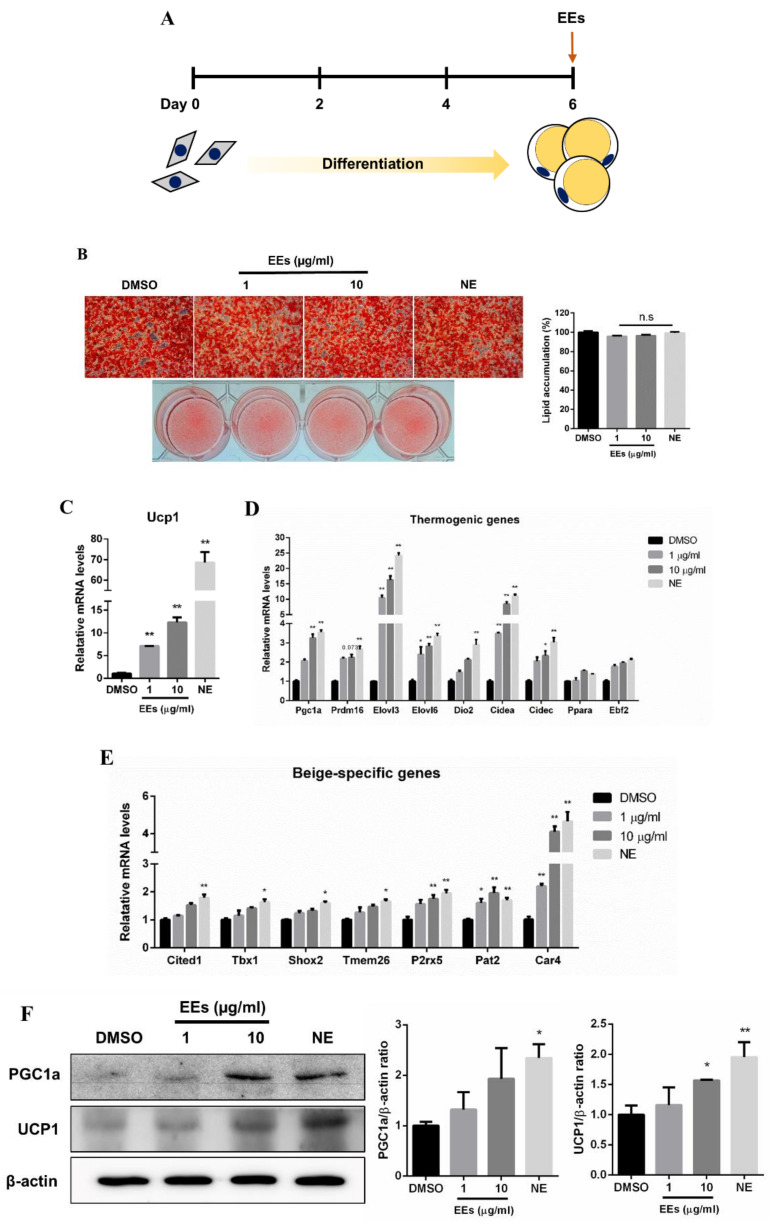
Effects of EEs on the browning process in differentiated mIPA. (**A**,**B**) Fully differentiated mIPA were treated with EEs for 6 h and stained with ORO dye. (**C**–**E**) Expression levels of *Ucp1*, thermogenic genes, and beige-specific genes in EEs-treated cells were investigated by real-time qPCR and compared with controls (DMSO and NE groups). (**F**) Protein levels of UCP1 and PGC1a were analyzed by Western blotting. Results are shown as mean ± SEM (*n* = 3). *, *p* < 0.05; **, *p* < 0.01 compared to the untreated control group.

Fully differentiated mIPA were treated with EEs for 6 h in order to confirm the browning potential of EEs on white adipocytes (Figure 2A). Norepinephrine (NE) was used as a positive control to induce browning of white adipocytes. In ORO staining, no difference was observed between the EEs-treated group, including the NE-treated group, and the untreated control group (Figure 2B), and the result of EEs treatment during differentiation of mIPA was not followed (Figure 1B). However, the mRNA expression of Ucp1 increased approximately 12~16-fold in EEs-treated group compared to the untreated control group (Figure 2C, *p* < 0.01). In addition, the protein level of Ucp1 was also significantly increased (~1.6-fold) in the 10 μg/mL EEs-treated group (Figure 2F, *p* < 0.01). Expression levels of thermogenic genes, including *Pgc1a* (~3.6-fold), *Elovl3*, *Elovl6, Cidea* (*p* < 0.01), and *Cidec* (*p* < 0.05), were significantly elevated by EEs treatment (Figure 2D). Pgc1a was also elevated at the protein level (Figure 2F, *p* < 0.01). EEs also up-regulated the expression of beige-specific genes, including *P2rx5*, *Pat2*, and *Car4* (~4.1-fold) (*p* < 0.01). In contrast, significant differences in expression levels of *Tmem26*, *Cited1*, *Tbx1*, and *Shox2* genes were not detected, although they showed an increasing trend in the group treated with 10 μg/mL EEs (Figure 2E). These results showed that treatment with 10 μg/mL EEs significantly promoted the expression of thermogenic and beige-specific genes, including *Ucp1*, in differentiated mIPA.

### 3.3. EEs Promotes Mitochondrial Activity and Biogenesis in Differentiated mIPA

**Figure 3:**
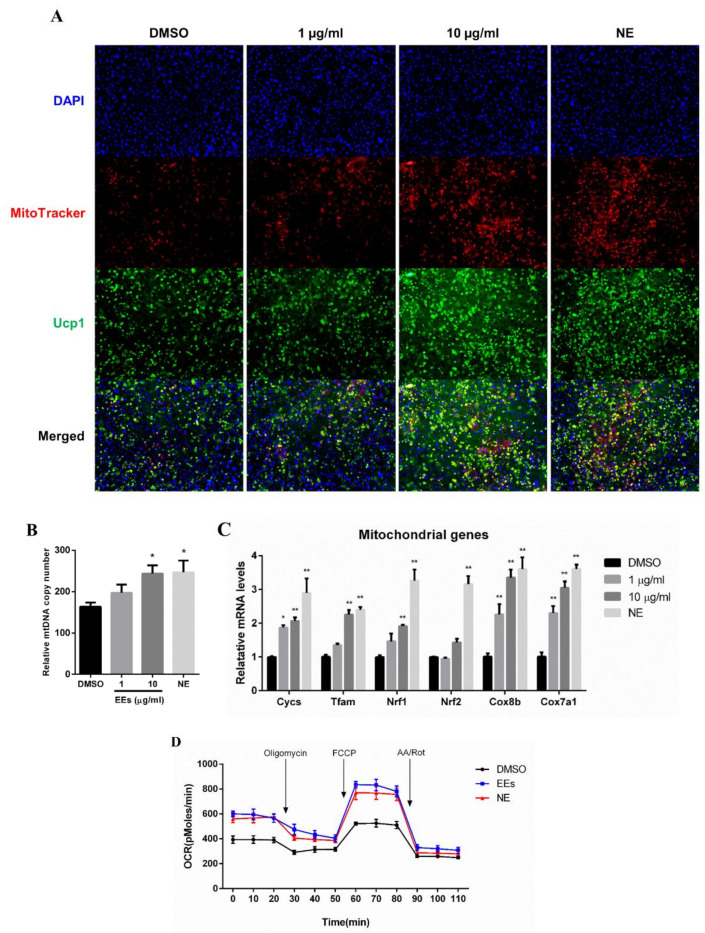
Effects of EEs on mitochondrial biogenesis and activity of differentiated mIPA. (**A**) Fully differentiated mIPA were stained with MitoTracker Red and anti-Ucp1 antibody, respectively. Images were analyzed with a fluorescence microscope. (**B**,**C**) Relative mtDNA copy number and expression levels of mitochondrial genes were analyzed by real-time qPCR. (**D**) Oxygen consumption rate of differentiated mIPA stimulated with 10 µg/mL of EEs and NE. Results are shown as mean ± SEM (*n* = 3). *, *p* < 0.05; **, *p* < 0.01 compared to the untreated control group.

To demonstrate whether enhanced thermogenesis in differentiated mIPA was caused by EEs treatment, mitochondrial activity was investigated. The mitochondrial content was increased in EEs-treated adipocytes compared to the untreated control group, as evidenced by fluorescence-stained mitochondria and immunofluorescence-stained mitochondrial-localized UCP1 (Figure 3A). Additionally, it was found that 10 μg/mL EEs up-regulated the expression of mitochondrial-related genes, including *Cycs*, *Tfam*, *Nrf1*, *Cox7a1* (~3-fold), *Cox8b* (~3.3-fold) (*p* < 0.01), and *Nrf2* and mtDNA copy number (*p* < 0.05) (Figure 3B,C). These results showed that EEs promoted fluorescence-stained mitochondria contents, mtDNA copy number and mitochondrial gene expression in differentiated mIPA.

### 3.4. EEs Stimulates Cellular Respiration Rate in Differentiated mIPA

Because EEs promoted mitochondrial biogenesis and the expression of thermogenesis-related genes in differentiated mIPA (Figure 3), an XF assay kit was used to assess whether EEs stimulated cellular respiration in matured white adipocytes. It was found that the OCR was increased in the EEs-treated group compared to the untreated control group (Figure 3D). Moreover, basal respiration, maximal respiration (*p* < 0.01), ATP production, and proton leak (*p* < 0.05), calculated by area under the curve (AUC), were also significantly up-regulated in the EEs-treated group compared to the untreated control group (Figure S2A). These results showed that treatment with EEs promoted cellular respiration in differentiated mIPA.

### 3.5. EEs Promotes Browning in Differentiated hADSCs

**Figure 4:**
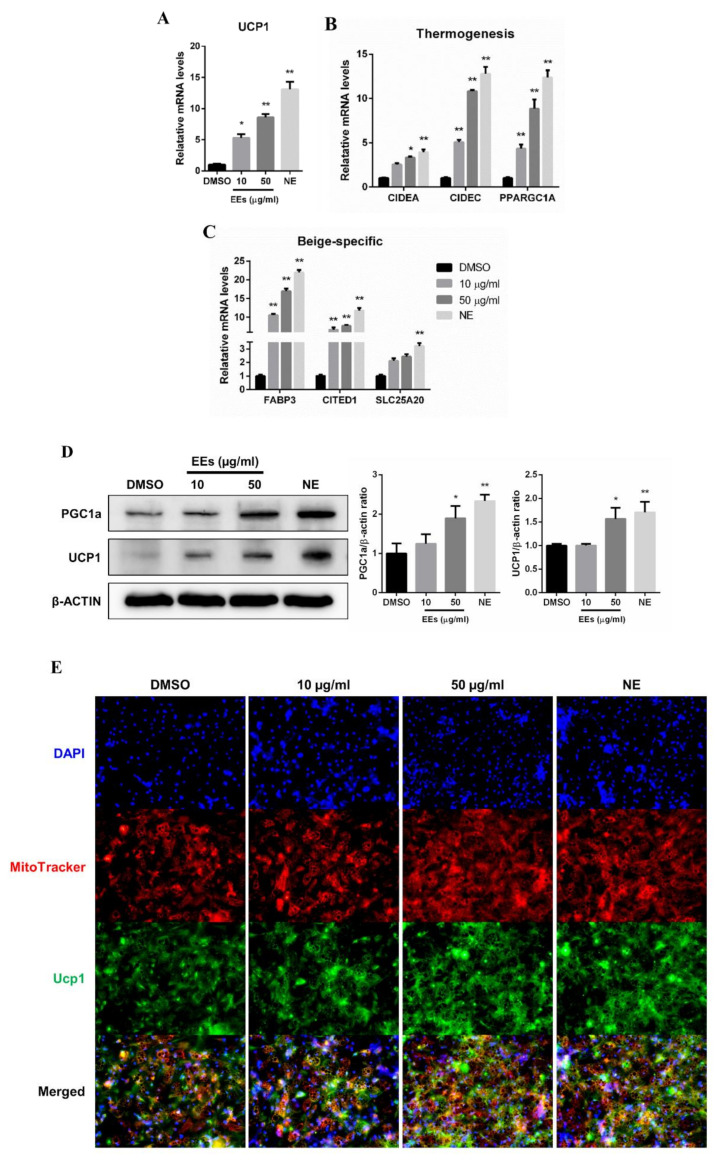

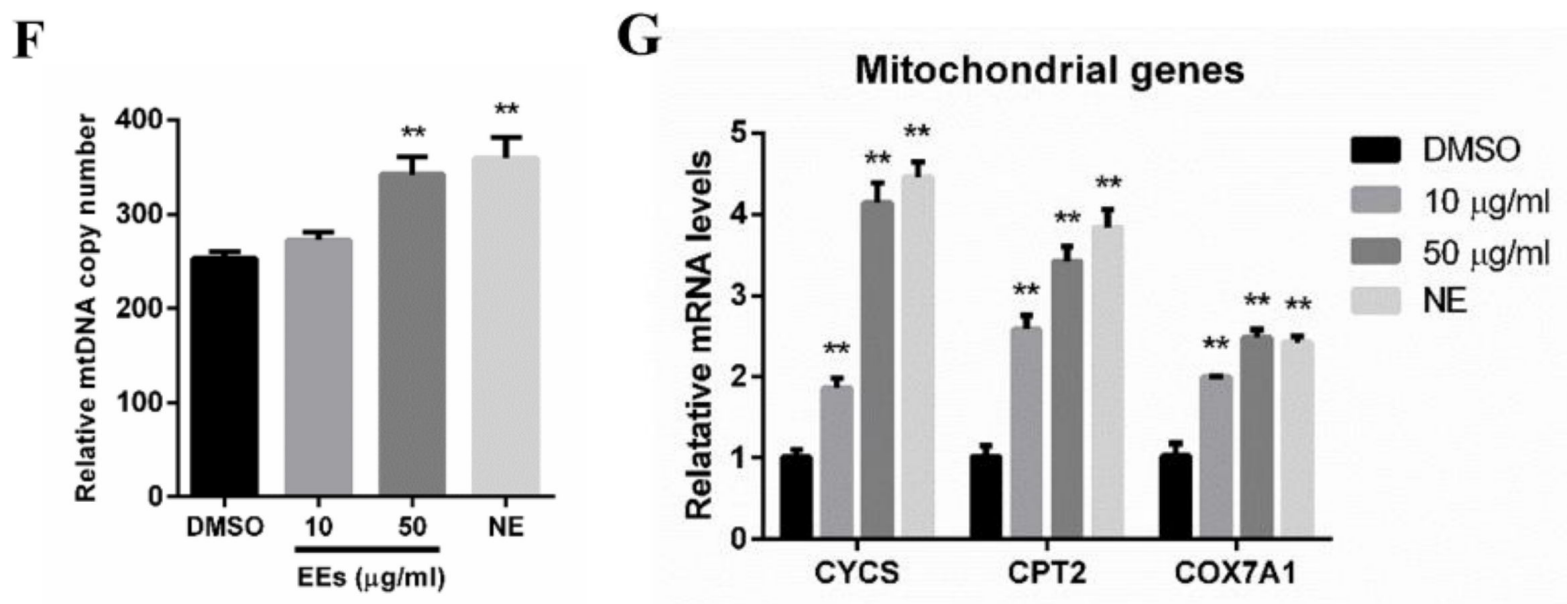
Effects of EEs on the browning process of differentiated hADSCs. (**A**–**C**) Relative mRNA levels of browning related genes were studied by real-time qPCR. (**D**) Protein levels of UCP1 and PGC1a were analyzed by Western blotting. (**E**) Cellular mitochondria and UCP1 were stained with MitoTrack Red and anti-UCP1 antibody, respectively. (**F**,**G**) Relative mtDNA copy number and expression levels of mitochondrial genes were analyzed by real-time qPCR. Results are shown as mean ± SEM (*n* = 3). *, *p* < 0.05; **, *p* < 0.01 compared to the untreated control group.

Effects of EEs on mouse adipocytes were compared with those on human adipocytes using hADSCs. In contrast to mIPA, 10 and 50 µg/mL of EEs in hADSCs showed non-cytotoxic, based on cell viability assay (Figure S1B). Cells were treated with EEs, NE, or DMSO for 6 h after completion of hADSCs differentiation. No significant difference in lipid accumulation was found (Figure S3A). Consistently, mRNA and protein levels of UCP1 in hADSCs were significantly enhanced by EEs in a dose-dependent manner (~8.6-fold and ~1.5-fold) (Figure 4A,D,E, *p* < 0.01), similar to results in mIPA. Next, expression levels of thermogenic, beige-specific, and mitochondrial factors in differentiated hADSCs were assessed. It was found that 50 µg/mL EEs treatment significantly up-regulated the expression of thermogenic genes, including *CIDEC*, *PPARGC1A* (~8.8-fold) (*p* < 0.01), and *CIDEA* (*p* < 0.05) (Figure 4B,D). EEs also enhanced mRNA expression levels of beige-specific genes, including *FABP3* (~16.9-fold), *CITED1* (*p* < 0.01) and *SLC25A20*, in the 50 µg/mL EEs-treated group compared to the untreated control group (Figure 4C). Furthermore, compared to the untreated control group, 50 µg/mL EEs treatment significantly promoted immunofluorescence staining of the mitochondria (Figure 4E), the mtDNA copy number (Figure 4F, *p* < 0.01), and the expression of mitochondrial-related genes, including *CYCS* (~4.1-fold), *CPT2* and *COX7A1* (~2.5-fold) (Figure 4G, *p* < 0.01). Overall, differentiated hADSCs with EEs treatment not only up-regulated thermogenic genes, including UCP1 and beige-specific genes, but also enhanced mitochondrial-related genes, fluorescence-stained mitochondria contents, and mtDNA copy number, as shown in mIPA.

## 4. Discussion

In terms of energy homeostasis between energy intake and expenditure, the relationship among three types of adipocytes plays a very important role in the treatment and prevention of obesity [24]. In particular, white adipocytes have various roles in energy homeostasis depending on the types of stimuli [4,5]. They have a role in energy storage under normal conditions. However, they are converted into thermogenic adipocytes that consume energy through the browning process upon various external stimuli, such as cold exposure, dietary factors and pharmacological drugs, thereby being involved in energy expenditure [3,4,5]. In addition, many studies have reported that herbal extracts containing phytochemicals can improve obesity and related metabolic diseases via downregulation of adipogenesis and upregulation of thermogenesis [4,13,14].

*E. sinica* is used as a traditional medicine in Asian countries to treat inflammation disease, obesity, asthma, and arthritis. *E. sinica* contains ephedrine as a bioactive compound constituent with several physiological actions [15]. Lee et al. have reported that EEs can inhibit lipid accumulation and prevent adipogenesis in 3T3-L1 white preadipocytes with a weight loss effect in mice [19]. A similar result was observed in the present study. EEs treatment during mIPA differentiation suppressed lipid accumulation and the expression of adipogenic genes, including *Pparg* and *Cebpa* (Figure 1). This result indicates that EEs can inhibit adipocyte differentiation and prevent the increase in the number of white adipocytes. This is consistent with the study of Baba et al., showing that the accumulation of ^18^F-fluorodeoxyglucose (^18^F-FDG) is increased in the group treated with a mixture containing ephedrine due to the activation of thermogenesis in brown adipocytes, resulting in weight loss [20]. However, no studies have reported the effect of EEs on the browning of mature white adipocytes. Therefore, this study extended previous studies in order to investigate the effect of EEs on the browning process in mature white adipocytes in mice and humans.

A typical browning of white adipocytes shows a high expression of UCP1 located in the inner mitochondria membrane. Interestingly, mRNA and protein expression levels of UCP1 were significantly increased in the EEs-treated group compared to the untreated control group (Figure 2C,F and Figure 3A). In addition, thermogenic and beige-specific markers were identified, demonstrating the browning effect of EEs. Pgc1a and Prdm16 are transcription factors involved in maintaining the beige adipocytes phenotype, and regulating thermogenesis in the initialization of adipocyte browning [25]. It has been reported that the expression of genes, including *Elovl3*, *Elovl6*, *Dio2*, *Cidea*, *Cidec*, *Ppara*, and *Ebf2*, is associated with adipocyte thermogenesis [26]. In this study, expression levels of thermogenic-related markers, such as *Pgc1a*, *Elovl3*, *Elovl6*, *Cidea*, and *Cidec*, were significantly increased in EEs-treated group compared to the untreated control group, whereas *Prdm16*, *Dio2*, *Ppara*, and *Ebf2* only showed an increasing trend (Figure 2D). Moreover, the expression of beige-specific marker genes also showed a tendency to increase (Figure 2E), similar to the results of previous studies [26]. Effects of EEs on thermogenic and beige-specific markers were also similarly found in human adipocytes (Figure 4). These data suggest that EEs has the dual roles of inhibiting the differentiation of white adipocytes while promoting the browning of white adipocytes. Furthermore, ephedrine is known to be an adrenergic receptor agonist to activate adrenergic receptor downstream effectors, including adenosine monophosphate-activated protein kinase (AMPK) and p38 mitogen-activated protein kinase (p38 MAPK) [27,28,29]. It has been reported that ephedrine is a primary factor in *E. sinica* and was sufficiently observed in the water extraction of the *E. sinica* used in this study [15]. These data imply that EEs is associated with the adrenergic receptor cascade to induce adipocyte browning.

Mitochondria are essential organelles for maintaining adipocyte function in metabolic homeostasis [30,31]. The browning of white adipocytes is associated with mitochondrial biogenesis and activity [31,32]. Beige and brown adipocytes have many mitochondria and a high expression of UCP1. In addition, activation of mitochondria in brown and beige adipocytes can increase UCP1 expression and promote thermogenesis [30,31]. In particular, *Pgc1a* was increased by EEs treatment in this study (Figure 2D). Pgc1a is a key transcriptional coactivator that promotes mitochondrial biogenesis [32]. Activation of Pgc1a can promote the expression of mitochondrial biogenesis and activity-related transcription factors, such as Tfam and Nrf1 [32]. They can promote the expression of mitochondrial activity-related genes, such as *Cycs*, *Cox8b* and *Cox7a1* [3]. All these reports suggest that EEs treatment is related to the increased mitochondrial activity (Figure 3C). Furthermore, to better understand the effect of EEs on mitochondrial biogenesis, mitochondrial activity in cells was evaluated based on immunofluorescence and mtDNA copy number. It was confirmed that the number of stained mitochondria and mtDNA copies were increased in the EEs-treated group compared to the untreated control group (Figure 3A,B). This indicates that treatment with EEs can promote mitochondrial biogenesis in white adipocytes, thereby increasing the number of mitochondria. In addition, the increase in cellular respiration of differentiated mIPA induced by the EEs observed in this study supports the findings that mitochondrial biogenesis and upregulation of activity are associated with increased mitochondria contents (Figure 3). These data indicate that EEs can regulate the number of mitochondria and promote energy expenditure.

In conclusion, this study demonstrated that EEs could inhibit the differentiation of white adipocytes, promote the browning of mature adipocytes, and increase the energy consumption rate by regulating the number of mitochondria in both mouse and human primary adipocytes. The results of this study suggest that *E. sinica* is an attractive and realistic herbal extract for regulating energy metabolism and treating obesity due to its efficacy in increasing energy expenditure through white adipocyte browning.

## Data Availability

All raw data used for figure generation in this manuscript can be obtained by contacting the corresponding author.

## Supplementary Materials

The following supporting information can be downloaded at: https://www.mdpi.com/article/10.3390/foods11071028/s1, Figure S1: Cytotoxic effects of EEs on mIPA and hADSCs. (A) Cell viability assay performed on mIPA treated with EEs in a dose-dependent manner. (B) Cell viability assays were performed for hADSCs treated with various dosages of EEs. Results are shown as mean ± SEM (*n* = 3). *, *p* < 0.05; **, *p* < 0.01 compared with the untreated control group; Figure S2: Effects of EEs on cellular respiration rate of differentiated mIPA. (A) Basal respiration, ATP production, proton leak, and maximal respiration were assessed. Results are shown as mean ± SEM (*n* = 3). *, *p* < 0.05; **, *p* < 0.01 compared with the untreated control group; Figure S3: Effects of EEs on lipid accumulation of differentiated hADSCs. (A) ORO staining performed after EEs treatment. Results are shown as mean ± SEM (*n* = 3). *, *p* < 0.05; **, *p* < 0.01 compared with the untreated control group; Table S1: Primer sequences of quantitative PCR (5′–3′).

## Abbreviations

CAR4, carbonic anhydrase 4; CEBPa, CCAAT/enhancer binding protein alpha; FABP4, fatty acid binding protein 4; CIDEA, cell death-inducing DNA fragmentation factor, alpha subunit-like effector A; CIDEC, cell death-inducing DFFA-like effector C; CITED1, Cbp/p300-interacting transactivator with Glu/Asp-rich carboxy-terminal domain 1; COX7A1, cytochrome c oxidase subunit 7A1; COX8B, cytochrome c oxidase subunit 8B; CPT2, carnitine palmitoyltransferase 2; CYCS, cytochrome c; DIO2, deiodinase, iodothyronine, type II; EBF2, early B cell factor 2; TMEM26, transmembrane protein 26; ELOVL3, elongation of very long-chain fatty acid-like 3; ELOVL6, ELOVL family member 6; FABP3, fatty acid binding protein 3; NRF1, nuclear respiratory factor 1; NRF2, nuclear factor, erythroid derived 2, like 2; P2RX5, purinergic receptor P2X, ligand-gated ion channel, 5; PAT2, proton-coupled amino acid transporter 2; PGC1a, PPAR gamma coactivator 1 alpha; PPARa, peroxisome proliferator-activated receptor alpha; PPARg, peroxisome proliferator activated receptor gamma; PRDM16, PR domain containing 16; SHOX2, short stature homeobox 2; SLC25A20, solute carrier family 25 member 20; TBX1, T-box 1; TFAM, mitochondrial transcription factor A.
