# Supplementary material for: Extract of Ephedra sinica Stapf Induces Browning of Mouse and Human White Adipocytes
Source: Foods. 2022 Apr 1;11(7):1028. doi: 10.3390/foods11071028 (PMC8998140; doi:10.3390/foods11071028)
Supplement: Supplementary file 1 [file foods-11-01028-s001.zip › foods-1643665-supplementary.pdf]

Supplemental Table S1

Table S1. Primer sequences of quantitative PCR (5'-3')

Mouse	Forward	Reverse	Accession no.
Tbp	AACATCTCAGCAACCCACACA	CTGGTGTGGCAGGAGTGATAG	NM_013684
Pparg	TCCATTCACAAGAGCTGACCC	GGCCATGAGGGAGTTAGAAGG	NM_001127330
Cebpa	TGGACAAGAACAGCAACGAGT	ACCTTCTGTTGCGTCTCCAC	NM_001287514
Fabp4	GCTTTGCCACAAGGAAAGTG	ACGCCCAGTTTGAAGGAAAT	NM_024406
Ucp1	AGGATTGGCCTCTACGACTCA	GCATTCTGACCTTCACGACCT	NM_009463
Pgc1a	TCTGGGTGGATTGAAGTGGTG	TCTGTGAGAACCGCTAGCAAG	NM_008904
Prdm16	ACAAGTCCTACACGCAGTTC	CGTGTAATGGTTCTTGCCCCT	NM_001177995
Elovl3	CGTAAGCGTCCACTCATCTT	AGAATGGACGCCAAAGTTCA	NM_001374665
Elovl6	CCATCCCCAGATGCTTTGAA	GGATTTGGCTTTGCTTGACC	NM_130450
Dio2	GTGAGCTGGGAAAGTTGCTA	AAGAATAGAGCCGGTGCTTG	NM_010050
Cidea	AAATGGACACCGGGTAGTAAG	TTTGACATTGAGACAGCCGA	NM_007702
Cidec	GGCCTGAATCAAGACAACCA	GAGTGATCAGAGTCCCAGGT	NM_001301295
Ppara	GAACAAAGACGGGATGCTGAT	CAGCCACAAACAGGGAAATG	NM_001113418
Ebf2	CTACGGCACACCACACAATAA	TGATACCCATCATACCGCTGT	NM_001276387
Cited1	ATTTATCGGACTTCTGCCCAG	TTGCGATCCTTCACTCCAAG	NM_001276466
Tbx1	TGGGACGAGTTCAATCAGC	TGTCATCTACGGGCACAAAG	NM_001285472
Shox2	CCCACTATCCAGACGCTTTC	ATAGGGTGCAACTCTACAAGC	NM_001302357
Tmem26	CTCTGGTCTCTGGCATTCTTG	CATAGATCCGCACTGTACTGG	NM_177794
P2rx5	ACTTTGTCATTCCGTCTCAGG	CAACAGACTCCCCAGCGTGACA	NM_001376982
Pat2	ATGTCTGTGACCAAGAGTGC	CCAGGATCCCAGTCCAGCGAATG	NM_153170
Car4	GTAGGAGACAAGGTGAACAAGG	AGTTTGGTGTGGTTAGTGAGC	NM_007607
Cycs	AAATCTCCACGGTCTGTTCG	GTCTGCCCTTTCTCCCTTC	NM_007808
Tfam	GGAATGTGGAGCGTGCTAAAA	TTGGGTAGCTGTTCTGTGGAA	NM_009360
Nrf1	AAGATGCTAATGGCCTGGTCC	ATATCCTGGTGGTCACTGGGG	NM_001164226
Nrf2	CGCTGAAGGCACAATGGAATT	GCTTTAGGGCCGTTCTGTTTG	NM_001399226
Cox8b	AAAGCCCATGTCTCTGCCAA	GCTAAGACCCATCCTGCTG	NM_007751
Cox7a1	AGAAAACCGTGTGGCAGAGA	CCAGCCCAAGCAGTATAAGC	NM_009944
**Human**	**Forward**	**Reverse**	**Accession no.**
GAPDH	GGAAGGTGAAGGTCGGAGTC	GAAGGGGTCATTGATGGCAAC	NM_001256799
UPC1	ACTTGGTGTCGGCTCTTATCG	CCGTTGGTCCTTCGTTAGTGA	NM_021833
PPARGC1A	ACCCACCACTCCTCCTCATAA	GTCTTCCTTTCCTCGTGTCCA	NM_001330751
CIDEA	TGGGAGACAACACGCATTTCA	TCATACATGGTGGCCTTCACG	NM_001279
CIDEC	AAGTCCCTTAGCCTTCTCTACC	CCTTCCTCACGCTTCGATCC	NM_001199551

CITED1	CCTCACCTGCGAAGGAGGA	GGAGAGCCTATTGGAGATCCC	NM_001144885
FABP3	ACCAAGCCTACCACAATCATCG	CAAGTTTCCCTCCATCCAGTGT	NM_001320996
SLC25A20	AGACACAGCCACCGAGTTTG	TCCCCAAACCAAACCCAAAGA	NM_000387
CPT2	CAGCAGCCAAAGGGATCATCT	TACCCAACACCAAAGCCATCA	NM_000098
CYCS	TGGGTGATGTTGAGAAAGGCA	TGGCGGCTGTGTAAGAGTATC	NM_018947
COX7A1	GAGTGCGCGAGAAACAGAAG	CCCAGCCAAGGGAGTACAAG	NM_001864

Supplemental Figure S1

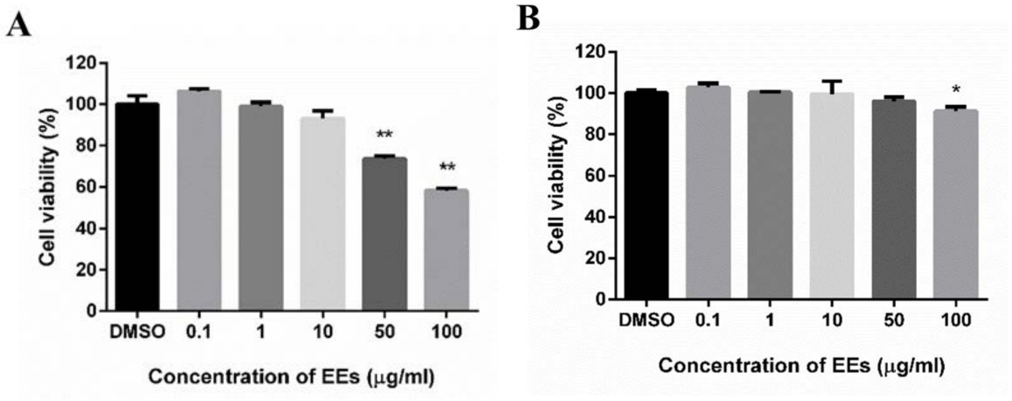

Figure S1. Cytotoxic effects of EEs on mIPA and hADSCs. (A) Cell viability assay performed on mIPA treated with EEs in a dose-dependent manner. (B) Cell viability assays were performed for hADSCs treated with various dosages of EEs. Results are shown as mean ± SEM (n = 3). *, $p < 0.05$; **, $p < 0.01$ compared with the untreated control group.

Supplemental Figure S2

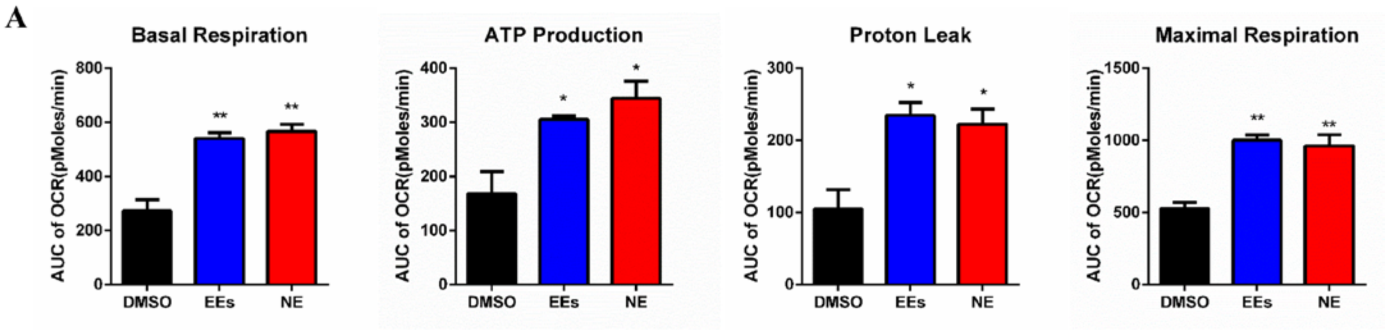

Figure S2. Effects of EEs on cellular respiration rate of differentiated mIPA. (A) Basal respiration, ATP production, proton leak, and maximal respiration were assessed. Results are shown as mean ± SEM (n = 3). *, $p < 0.05$; **, $p < 0.01$ compared with the untreated control group.

Supplemental Figure S3

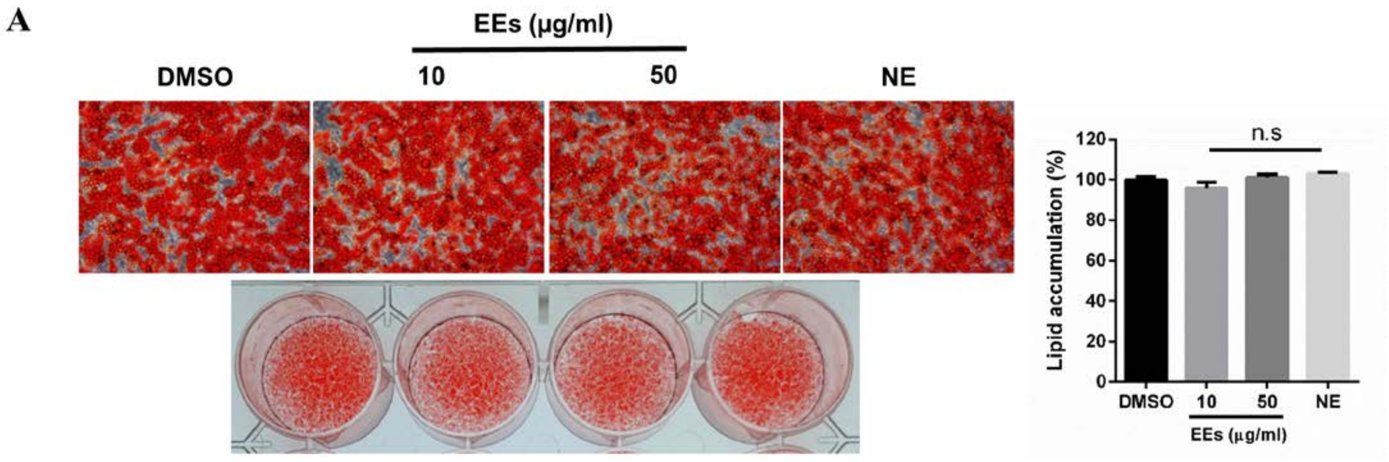

Figure S3. Effects of EEs on lipid accumulation of differentiated hADSC. (A) ORO staining performed after EEs treatment. Results are shown as mean ± SEM (n = 3). *, $p < 0.05$; **, $p < 0.01$ compared with the untreated control group.
